# Functional genomics identifies specific vulnerabilities in PTEN-deficient breast cancer

**DOI:** 10.1186/s13058-018-0949-3

**Published:** 2018-03-22

**Authors:** Yew Chung Tang, Szu-Chi Ho, Elisabeth Tan, Alvin Wei Tian Ng, John R. McPherson, Germaine Yen Lin Goh, Bin Tean Teh, Frederic Bard, Steven G. Rozen

**Affiliations:** 1 Programme in Cancer and Stem Cell Biology, Duke-NUS Medical School, 8 College Road, Singapore, 169857 Singapore; 2 Centre for Computational Biology, Duke-NUS Medical School, 8 College Road, Singapore, 169857 Singapore; 3 Institute of Molecular and Cell Biology, 61 Biopolis Drive, Singapore, 138673 Singapore; 4 National Cancer Centre Singapore, 11 Hospital Drive, Singapore, 169610 Singapore; 5 NUS Graduate School for Integrative Sciences and Engineering, National University of Singapore, 5 Lower Kent Ridge Road, Singapore, 119074 Singapore

**Keywords:** Synthetic lethality, Synthetic sickness, Precision medicine, PTEN, Breast cancer, Targeted cancer therapy, NUAK1, STK11, LKB1

## Abstract

**Background:**

Phosphatase and tensin homolog (PTEN) is one of the most frequently inactivated tumor suppressors in breast cancer. While PTEN itself is not considered a druggable target, PTEN synthetic-sick or synthetic-lethal (PTEN-SSL) genes are potential drug targets in PTEN-deficient breast cancers. Therefore, with the aim of identifying potential targets for precision breast cancer therapy, we sought to discover PTEN-SSL genes present in a broad spectrum of breast cancers.

**Methods:**

To discover broad-spectrum PTEN-SSL genes in breast cancer, we used a multi-step approach that started with (1) a genome-wide short interfering RNA (siRNA) screen of ~ 21,000 genes in a pair of isogenic human mammary epithelial cell lines, followed by (2) a short hairpin RNA (shRNA) screen of ~ 1200 genes focused on hits from the first screen in a panel of 11 breast cancer cell lines; we then determined reproducibility of hits by (3) identification of overlaps between our results and reanalyzed data from 3 independent gene-essentiality screens, and finally, for selected candidate PTEN-SSL genes we (4) confirmed PTEN-SSL activity using either drug sensitivity experiments in a panel of 19 cell lines or mutual exclusivity analysis of publicly available pan-cancer somatic mutation data.

**Results:**

The screens (steps 1 and 2) and the reproducibility analysis (step 3) identified six candidate broad-spectrum PTEN-SSL genes (*PIK3CB*, *ADAMTS20*, *AP1M2*, *HMMR*, *STK11*, and *NUAK1*). *PIK3CB* was previously identified as PTEN-SSL, while the other five genes represent novel PTEN-SSL candidates. Confirmation studies (step 4) provided additional evidence that *NUAK1* and *STK11* have PTEN-SSL patterns of activity. Consistent with PTEN-SSL status, inhibition of the NUAK1 protein kinase by the small molecule drug HTH-01-015 selectively impaired viability in multiple PTEN-deficient breast cancer cell lines, while mutations affecting *STK11* and *PTEN* were largely mutually exclusive across large pan-cancer data sets.

**Conclusions:**

Six genes showed PTEN-SSL patterns of activity in a large proportion of PTEN-deficient breast cancer cell lines and are potential specific vulnerabilities in PTEN-deficient breast cancer. Furthermore, the NUAK1 PTEN-SSL vulnerability identified by RNA interference techniques can be recapitulated and exploited using the small molecule kinase inhibitor HTH-01-015. Thus, NUAK1 inhibition may be an effective strategy for precision treatment of PTEN-deficient breast tumors.

**Electronic supplementary material:**

The online version of this article (10.1186/s13058-018-0949-3) contains supplementary material, which is available to authorized users.

## Background

The discovery of specific vulnerabilities in phosphatase and tensin homolog (PTEN)-deficient cancers is clinically important because PTEN is one of the most frequently inactivated tumor suppressors in human cancer [1, 2]. Inactivation of PTEN can occur through loss-of-function genetic mutations, epigenetic silencing and transcriptional regulation, post-transcriptional regulation by non-coding RNAs, and through post-translational modifications and protein-protein interactions [3]. Germline *PTEN* mutations that result in loss of PTEN function confer an increased risk of developing benign and malignant tumors of the breast, thyroid, and endometrium [4]. Significantly, 67 to 85% of women with germline *PTEN* mutations develop breast cancer [5]. Although somatic *PTEN* mutations occur in only 5% of sporadic breast cancers, PTEN protein expression is significantly reduced in 25 to 37% of all breast tumors [6, 7]. PTEN loss in breast cancer is also associated with more aggressive disease and worse outcomes [8]. In particular, PTEN deficiency occurs more frequently in triple-negative breast cancers, which are not responsive to targeted cancer therapies [6, 8–11]. Therefore, the identification of specific vulnerabilities in PTEN-deficient breast cancer may suggest potential drug targets for an aggressive subset of breast cancers for which there is no effective therapy.

It has been challenging to clinically target PTEN-deficiency in cancer despite the well-established rationale for doing so. This is because PTEN function cannot directly be restored using small molecule drugs. The best-characterized function of PTEN is in antagonizing the phosphatidylinositol 3-kinase (PI3K)/AKT signaling pathway, which is essential for cell survival. PI3K activity is responsible for the formation of phosphatidylinositol (3,4,5)-trisphosphate (PIP_3_), a key second messenger that promotes phosphorylation and activation of the AKT kinase. AKT in turn phosphorylates and regulates multiple downstream processes. PTEN acts as a brake on this pathway by dephosphorylating PIP_3_ and suppressing AKT activation [1]. Consequently, loss of PTEN function removes a molecular brake on this pathway and allows PI3K to unabatedly activate downstream AKT signaling, thereby promoting cell survival and tumor formation [12]. As PTEN is a well-characterized PI3K pathway regulator, most drugs currently in clinical development for the treatment of PTEN-deficient cancers are kinase inhibitors that attempt to compensate for the loss of PTEN by suppressing PI3K/AKT signaling [13]. However, PTEN regulates multiple cell processes, including growth, proliferation, survival, chromosome stability, and DNA damage repair through mechanisms that are both dependent and independent of the PI3K pathway [14]. Thus, it is our hypothesis that there are unidentified therapeutic opportunities among the largely unexplored, PI3K-independent vulnerabilities of PTEN-deficient breast cancers.

Some of these therapeutic opportunities may exist in the form of PTEN synthetic-sick or synthetic-lethal (PTEN-SSL) genes. Synthetic lethality and synthetic sickness are terms used to describe gene-gene interactions that result in reduced cell viability or fitness [15]. A synthetic lethal relationship exists between two genes if their simultaneous perturbation results in death. Analogously, there is a synthetic sick relationship if simultaneous perturbation leads to reduced growth. In the context of cancer therapy, synthetic sick/lethal (SSL) interactions are of potential importance if SSL partners of oncogenes or tumor suppressor genes can be identified. In such cases, targeting the SSL partner by small molecule inhibitors or RNA interference (RNAi) might inhibit tumor growth or survival. This is well illustrated in the treatment of BRCA-deficient cancers with poly(ADP-ribose) polymerase (PARP) inhibitors, which exploit synthetic lethality between the *PARP* and *BRCA* genes [16]. A PTEN-SSL gene is, by definition, essential for cell survival or proliferation in PTEN-deficient (PTEN-) cells but not in PTEN-expressing (PTEN+) cells. PTEN-SSL genes would thus constitute potential vulnerabilities that could be exploited for the treatment of PTEN-deficient breast cancer.

**Fig. 1:**
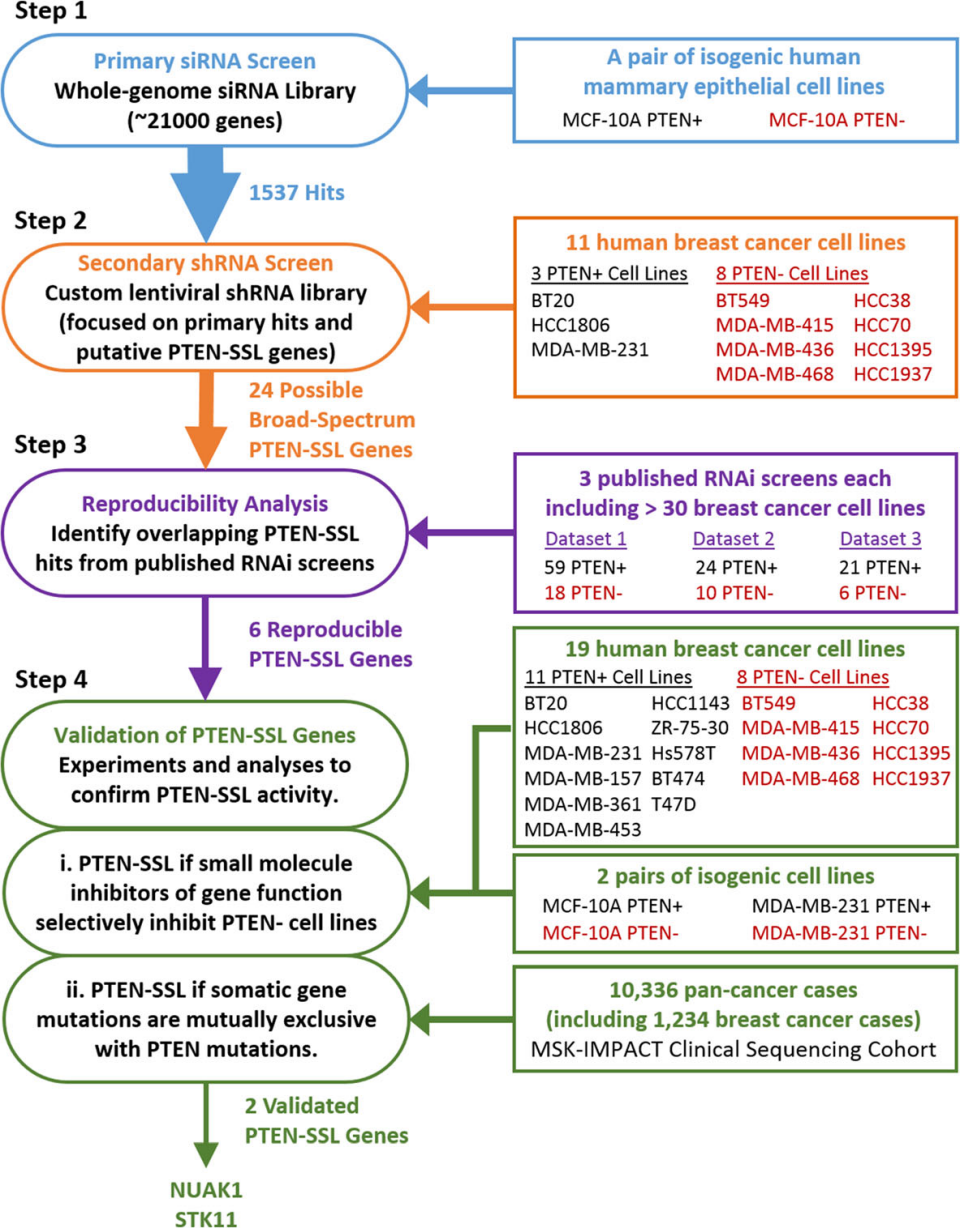
Overview of the phosphatase and tensin homolog-synthetic sick/lethal (PTEN-SSL) screening approach: the multi-step strategy used in this study to identify and validate broad-spectrum PTEN-SSL genes in breast cancer. siRNA, small interfering RNA; shRNA, short hairpin RNA; RNAi, RNA interference; NUAK1, NUAK family kinase 1; STK11, serine/threonine kinase 11

Here, we present the results of a multi-step strategy to identify PTEN-SSL genes in breast cancer (Fig. 1). We began with a two stage RNAi screening strategy of a large number of genes and followed up with reproducibility and confirmation studies of the most promising hits. First, to comprehensively detect a large set of possible PTEN-SSL genes, we carried out a PTEN-SSL screen of essentially all human genes (~ 21,000 genes) in an isogenic, non-malignant, breast epithelial cell line model. Second, to select genes with evidence of PTEN-SSL activity across multiple cell lines, we screened hits from the first screen combined with other previously reported PTEN-SSL candidate genes (~ 1200 genes total) in 11 genetically diverse breast cancer cell lines, of which 8 were PTEN-deficient. To identify candidate PTEN-SSL genes that have reproducible and general PTEN-SSL activity in breast cancer, we identified hit overlaps between our results and reanalyzed data from three publicly available gene-essentiality screens carried out in large panels of breast cancer cell lines [17–19]. Finally, where small molecule inhibitors of gene function or somatic gene mutation data in large tumor cohorts were available, we further investigated the PTEN-SSL activity of selected candidate genes. Namely, we assessed drug sensitivity in an expanded panel of 19 breast cancer cell lines and also looked for independent evidence of PTEN-SSL activity in the form of mutual exclusivity of mutations in large tumor cohorts.

## Methods

### Cell lines

We utilized a pair of isogenic PTEN+ and PTEN- MCF-10A cell lines (obtained from Horizon Discovery, HD 101–006) for our primary short interfering RNA (siRNA) screen. MCF-10A PTEN+ cells express the wild-type PTEN protein, while MCF-10A PTEN- cells were engineered to be PTEN-deficient [20]. The parental MCF-10A cell line was derived from benign proliferative breast tissue and shows normal expression of wild-type PTEN [21–24]. Both MCF-10A cell lines were cultured in DMEM/F-12 including 2.5 mM L-glutamine and 15 mM HEPES (Gibco), supplemented with 10% fetal bovine serum (Hyclone), 10 μg/ml insulin (Sigma), 20 ng/ml epidermal growth factor (Sigma), and 500 ng/ml hydrocortisone (Calbiochem) [25].

In addition to the MCF-10A cell lines, we used 19 human breast cancer cell lines in this study. Of these cell lines, 11 were screened with a short hairpin shRNA (shRNA) library and all 19 were tested in drug sensitivity experiments. The human breast cancer cell lines HCC38, HCC70, HCC1395, HCC1806, HCC1937, BT20, BT549, MDA-MB-231, MDA-MB-436, MDA-MB-468, and Hs578T were obtained directly from American Type Culture Collection (ATCC) while BT474, HCC1143, MDA-MB-157, MDA-MB-361, MDA-MB-415, MDA-MB-453, T47D, and ZR-75-30 were obtained through the Duke University Cell Culture Facility. Breast cancer cell lines were cultured using growth medium as previously reported (Additional file 1: Table S1) [26]. All cells were cultured in a humidified atmosphere at 37 °C in 5% CO_2_ and all cell culture medium was supplemented with 50 units/ml penicillin and 50 mg/ml streptomycin.

We generated the MDA-MD-231 PTEN+ and PTEN- isogenic cell lines used in drug sensitivity experiments by lentivirally transducing MDA-MB-231 cells with a non-silencing shRNA (Open Biosystems - GE Dharmacon, RHS4351) and a shRNA pool targeting human PTEN (Open Biosystems - GE Dharmacon, RHS4533-NM_000314), respectively. Lentivirus particles were produced in HEK293T cells using the Trans-Lentiviral pGIPZ Packaging System (Open Biosystems - GE Dharmacon, TLP4615) according to the manufacturer’s protocol. We selected for cells with viral integration using puromycin. We confirmed stable knockdown of PTEN protein in the MDA-MB-231 PTEN- cell line through western blotting: the MDA-MB-231 PTEN+ cell line expressed PTEN at wild-type levels while the MDA-MB-231 PTEN- cell line expressed lower PTEN protein levels.

### PTEN protein detection and quantification

PTEN protein abundance in 19 breast cancer cell lines was assayed using western blots. Cells were lysed with lysis buffer containing 2% SDS (sodium dodecyl sulfate) when they reached 80% confluence. Lysate protein concentration was determined using micro BCA assay (Pierce). The same amount of protein (15 μg) from each cell line was prepared in Laemmli sample buffer and loaded into each well of hand-cast 10% SDS-polyacrylamide gels. SDS-polyacrylamide gel electrophoresis (SDS-PAGE) and western blotting of protein to nitrocellulose membranes was carried out using the Mini-PROTEAN Tetra system (Bio-Rad) according to the manufacturer’s instructions. The resulting membranes were probed with antibodies targeting PTEN (Cell Signaling Technology, #9552) and Actin (Chemicon, MAB1501R) according to the manufacturers’ instructions. DyLight 800 conjugated goat anti-rabbit and anti-mouse secondary antibodies were used in visualization of protein bands using the LI-COR Odyssey infrared imaging system (LI-COR Biosciences). There were 8 cell lines (HCC38, HCC70, HCC1395, HCC1937, BT549, MCA-MB-415, MDA-MB-436, and MDA-MB-468) with no visible PTEN protein bands that were categorized as PTEN- while the other 11 cell lines with visible PTEN bands were considered PTEN+.

For quantification of protein abundance, we analyzed the western blot images using the Image Studio software (LI-COR) and protein bands were quantified using densitometric analysis. The densities of PTEN protein bands for each cell line were normalized to the actin protein bands (loading controls) from the same cell line. Two reference cell lines, one PTEN+ (HCC1806) and one PTEN- (BT549), were included on all gels/membranes and data from these cell lines were used to normalize protein quantities across membranes (Additional file 1: Table S2). We also obtained publicly available quantitative PTEN protein abundance data that were measured by reverse-phase protein arrays (RPPA) for 75 breast cancer cell lines [17], which included all 19 cell lines that we analyzed by western blot. We identified good correlation between RPPA-measured PTEN abundance values and our quantitative western blot measurements across cell lines, which were assayed using both methods (Spearman’s rho 0.87, *p* < 0.0001). Based on this correlation, we established a quantitative cutoff for identifying PTEN+ and PTEN- cell lines. We thus categorized 18 breast cancer cell lines as PTEN- and the remaining 57 cell lines as PTEN+ (Additional file 1: Table S3).

### Primary siRNA screen protocol

We carried out the primary siRNA screen in PTEN+ and PTEN- MCF-10A cell lines. Cell lines were screened using the whole-genome siGENOME siRNA library from Dharmacon (now Thermo-Fisher). The entire library, comprising 21,121 siRNA SMARTpools (pools containing 4 different siRNAs targeting each gene) was arrayed (one SMARTpool per well) in 68 black-walled 384-well plates (Greiner, #781091). Three types of control were included on all screening plates: non-silencing negative controls (48 wells containing Qiagen AllStars Negative Control siRNA; SI03650318), essential gene-positive controls (8 wells containing a siRNA pool targeting *PLK1*), and PTEN-SSL gene-positive controls (8 wells containing a siRNA pool targeting *CDC25A*). The screen was done by reverse transfecting 250 cells with 25 nM siRNA and 0.25 μl HiPerFect transfection reagent (Qiagen) in each well and then culturing the cells for 5 days. After 5 days, cells were fixed with 4% paraformaldehyde, permeabilized with 0.1% triton-x, and stained with Hoechst 33,342 (Molecular Probes). Stained nuclei were imaged and automatically counted using an ImageXpress Micro high-content imaging system (Molecular Devices) to generate raw cell counts after siRNA perturbation.

### Primary siRNA screen analysis

Percentage cell viability after siRNA perturbation was calculated as:

$$ {\left[\left(x\hbox{--} {\upmu}_{\mathrm{pos}}\right)/\left({\upmu}_{\mathrm{neg}}\hbox{--} {\mu}_{pos}\right)\right]}^{\ast }\ 100, $$

where *x* is the cell count for a given siRNA pool, *μ*_*neg*_ is the mean cell count for non-silencing negative controls (interpreted as indicating highest possible viability), and *μ*_*pos*_ is the mean cell count for essential-gene, positive controls (interpreted as indicating highest possible lethality) arrayed on each individual plate. Two replicate PTEN-SSL siRNA screens, each comprising parallel screens in PTEN+ and PTEN- cell lines, were carried out. We looked for PTEN-SSL hits by assessing the PTEN-dependent activity attributable to each siRNA SMARTpool. We first calculated the change in cell viability (∆viability) attributable to PTEN loss by subtracting PTEN- cell line viability from PTEN+ cell line viability. We then scored PTEN-SSL activity of a specific siRNA pool by calculating a *z* score based on the ∆viability of that siRNA relative to the ∆viability distribution (mean and standard deviation) of all 3264 non-silencing negative controls screened. Primary screen results (∆viability and *z* score data) can be found in Additional file 1: Table S4.

### Primary siRNA screen quality assessment

For screen quality assessment, we used “Z-prime factors”, as described previously [27], to determine how well separated the on-plate negative and positive controls were, thus estimating the dynamic range of each 384-well screening plate. Z-prime factors were calculated as:

$$ 1\hbox{--} {3}^{\ast }\ \left({\sigma}_{pos}+{\sigma}_{neg}\right)/\mid {\upmu}_{\mathrm{pos}}\hbox{--} {\upmu}_{\mathrm{neg}}\mid, $$

where *μ* and *σ* are the mean and standard deviation of the positive (pos) and negative (neg) control cell counts for each plate, respectively. A Z-prime factor between 0.5 and 1 would indicate an excellent assay while a Z-prime factor between 0 and 0.5 is marginally acceptable. A Z-prime factor <0 would indicate that the overlap between the positive and negative controls is too great for the assay to be useful. We used a threshold of Z-prime factor >0 to define acceptable screening plates, since RNAi assays with Z-prime factors >0 have been successful in identifying validated hits in duplicate or triplicate lethality screens [28]. When Z-prime factors were calculated using the essential-gene positive control that drastically inhibits cell viability in both cell lines, all plates screened passed quality assessment with a mean Z-prime factor of 0.70 across both cell lines and replicates. Similarly, all PTEN- cell line screening plates passed quality assessment when Z-prime factors were calculated using the PTEN-SSL positive control. Their mean Z-prime factor was 0.59. Thus, our primary screen had sufficient dynamic range to identify genes that had an impact on cell viability when they were knocked down in individual cell lines.

### Secondary shRNA screen protocol

To identify genes with broad-spectrum PTEN-SSL activity, we carried out a pooled lentiviral shRNA dropout viability screen in a panel of 11 breast cancer cell lines, of which 8 were PTEN- (HCC38, HCC70, HCC1395, HCC1937, BT549, MCA-MB-415, MDA-MB-436, and MDA-MB-468) and 3 were PTEN+ (BT20, HCC1806, MDA-MB-231). The custom shRNA library (Cellecta) comprised a total of 6500 individually barcoded lentiviral shRNA constructs targeting ~ 1200 genes (Additional file 1: Table S5). Genes targeted included ~ 1000 PTEN-SSL hits identified in the primary screen, together with 9 putative PTEN-SSL genes, which were reported in other studies but failed to score as hits in our primary screen. The remaining ~ 200 genes represent non-PTEN-SSL hits, which were included as controls. The custom-designed shRNA library included multiple shRNA constructs (5–10 individual shRNA sequences) to target each gene. The shRNA sequences were designed using Cellecta’s proprietary shRNA design algorithm and database of validated shRNAs, which have been optimized for RNAi genetic screens in pooled format (sequences provided in Additional file 1: Table S5). The library was constructed in the pRSI16-U6-(sh)-HTS6-UbiC-TagRFP-2A-Puro vector, and provided as pre-packaged ready-to-transfect VSV-g pseudotyped lentiviral particles by the manufacturer.

We infected cell lines with a single pool of lentiviral particles at a multiplicity of infection (MOI) between 0.1 and 0.5 and at 500–1000-fold representation of the library. Under these conditions, most cells were infected with either one or no shRNA constructs. Medium was replenished 24 h post infection. Infection efficiency was monitored using the percentage of virus-integrated red fluorescent protein positive (RFP+) cells tracked by fluorescence-activated cell sorting (FACS). We confirmed that MOI did not exceed 0.5 for all cell lines screened. At 2 days after infection, when viral integration was presumed complete, we exposed the cells to puromycin for 3 days to select for cells with viral integration. A start sample was collected from the population when puromycin selection was completed. Cells were subcultured when they reached 80% confluence to ensure continued logarithmic growth. Sufficient cells (determined by cell counting) were maintained in each passage so that on average there would be at least 500 cells per shRNA. We did this by seeding > 500 × 6500 = 3.25 million cells in each passage, with 6500 being the size of the library. When cells reached 4–6 population doublings, an end sample was taken. Genomic DNA was extracted from the start and end samples and sent to Cellecta for barcode amplification, next-generation sequencing, and enumeration of shRNA barcode counts from raw sequenced data (Additional file 1: Table S6).

### Secondary shRNA screen analysis

Screening data for all cell lines was provided by Cellecta as normalized shRNA-barcode counts adjusted to 20 M reads. To track changes in shRNA abundance over the course of the screen, we calculated the fold change in normalized barcode counts between the start and end of each screen (Additional file 1: Table S7). Since each gene was targeted by multiple shRNAs, we aggregated shRNA-level fold-change data into gene-level viability scores using the ATARiS (analytic technique for assessment of RNAi by similarity) algorithm [29]. ATARiS analyzes data from multiple shRNAs targeting each gene across all cell lines to select shRNAs with consistent activity profiles across all cell lines (Additional file 1: Table S8). ATARiS then uses the shRNAs with consistent profiles to calculate the gene-level viability score for each gene in each cell line (Additional file 1: Table S9). We ran the ATARiS version 2 algorithm using the GenePattern public server [30]. ATARiS sometimes generates > 1 solution per gene depending on the number of shRNA clusters showing the same activity profile across cell lines. We used all gene solutions generated by ATARiS. To detect broad-spectrum PTEN-SSL genes, we analyzed differences in cell line sensitivity attributable to PTEN loss by comparing the gene-level ATARiS scores of PTEN- cell lines against those of PTEN+ cell lines.

### Reanalysis of data from previous, independent RNAi screens

We assessed the reproducibility of our PTEN-SSL hits through a reanalysis of three independent, previously published RNAi screens. We accessed sensitivity scores reported for each study. Per-gene zGARP scores were downloaded from the Breast Functional Genomics study website (http://neellab.github.io/bfg/) [17] and these are available in Additional file 1: Table S11. DEMETER *z* scores for the Cancer Dependency Map dataset (Achilles v2.20.2 gene solutions) were downloaded from the Project Achilles website (https://portals.broadinstitute.org/achilles/datasets/all) [19] and are available in Additional file 1: Table S12. The *z* scores for the Kinase Dependency Profiles dataset were downloaded from Table S1 of the published study [18] and are available in Additional file 1: Table S13. We identified overlapping hit genes and tested the statistical significance of observing a given overlap between two sets of genes using the hypergeometric distribution (R function dhyper). The hypergeometric *p* value reflects the probability that an overlap of the observed cardinality or greater will occur as a result of randomly picking two sets of genes of the given hit-list sizes from the pool of all genes present in both datasets [31]. Overlaps with a *p* value <0.05 were considered statistically significant.

### Drug sensitivity experiments

We treated breast cancer cell lines with NUAK inhibitors WZ4003 (S7317) and HTH-01-015 (S7318), which were purchased from Selleck Chemicals and diluted in dimethyl sulfoxide (DMSO). For determination of cell line drug sensitivity, 1000–3000 cells were seeded in each well of 96-well plates and treated with 1–30 μM of drug. Cell viability was measured using a MTS cell proliferation assay (Promega CellTiter AQueous One) when the cells reached 80–90% confluence 3–5 days after treatment. The surviving fraction of cells in each well was determined relative to DMSO-treated controls. Drug sensitivity experiments were carried out in quadruplicate. Drug sensitivity was determined by calculating the drug concentration required to reduce cell viability by 50% relative to DMSO-treated controls (IC_50_). IC_50_ values were determined from dose-response data using GraphPad Prism version 7.

### Mutual exclusivity analysis of genetic alterations in patient cohorts

To look for patterns of mutual exclusivity between PTEN and candidate PTEN-SSL genes, we analyzed a breast cancer dataset (The Cancer Genome Atlas (TCGA) provisional breast invasive carcinoma cohort [6, 32]) and a pan-cancer patient dataset (the MSK-IMPACT study [33]). For each pair of genes, cBioPortal [34, 35] computes an “odds-ratio” for co-occurrence versus mutual exclusivity and a Fisher’s exact test *p* value of statistical significance. The “odds ratio” for two genes, *G1* and *G2*, is computed as “(*A* * *D*) / (*B* * *C*), [w]here *A* = number of cases altered in both genes; *B* = number of cases altered in *G1* but not *G2*; *C* = number of cases altered in *G2* but not *G1*; and *D* = number of cases altered in neither genes (sic)” (quoted verbatim from [34]). We used the criteria of odds ratio <1 and *p* < 0.05 to identify mutually exclusive mutation patterns.

### Statistical analysis

Data with assumed equal variance were analyzed by the *t* test and this was computed using GraphPad Prism version 7. For the secondary screen and the reanalysis in step 3, we used the one-sided Wilcoxon rank-sum test (R function wilcox.test) for comparing ATARiS scores between two groups of cell lines (PTEN+ and PTEN-). We used this non-parametric test because the ATARiS scores in our secondary screen and the sensitivity measures in step 3 had non-normal distributions (all *p* < 0.0001 using the D’Agostino-Pearson omnibus normality test in GraphPad Prism) and were compared in the same way. The alternative hypothesis was that the median of the PTEN- cell line ATARiS scores is lower than that of PTEN+ cell line scores.

## Results

### Primary siRNA screen in isogenic cell lines

To identify PTEN-SSL genes in breast cancer, we first carried out a primary small interfering RNA (siRNA) screen in a pair of isogenic cell lines (Step 1 in Fig. 1). These were derived from the MCF-10A human mammary epithelial cell line and differed only in PTEN status [20]. MCF-10A is an immortalized, but non-tumorigenic, breast epithelial cell line with a basal-like gene expression profile [21, 22]. Wild-type MCF-10A cells (MCF-10A PTEN+) express PTEN protein, while the PTEN knockout cells (MCF-10A PTEN-) do not. We chose a non-tumorigenic cell line for screening based on the hypothesis that PTEN-SSL interactions that are independent of oncogenic alterations would more likely generalize to a broad range of PTEN- tumors. We further hypothesized that, although some of the PTEN-SSL activities detected in the primary screen might be restricted to the MCF-10A cell line, our secondary screen in multiple cell lines would filter out such restricted vulnerabilities.

**Fig. 2:**
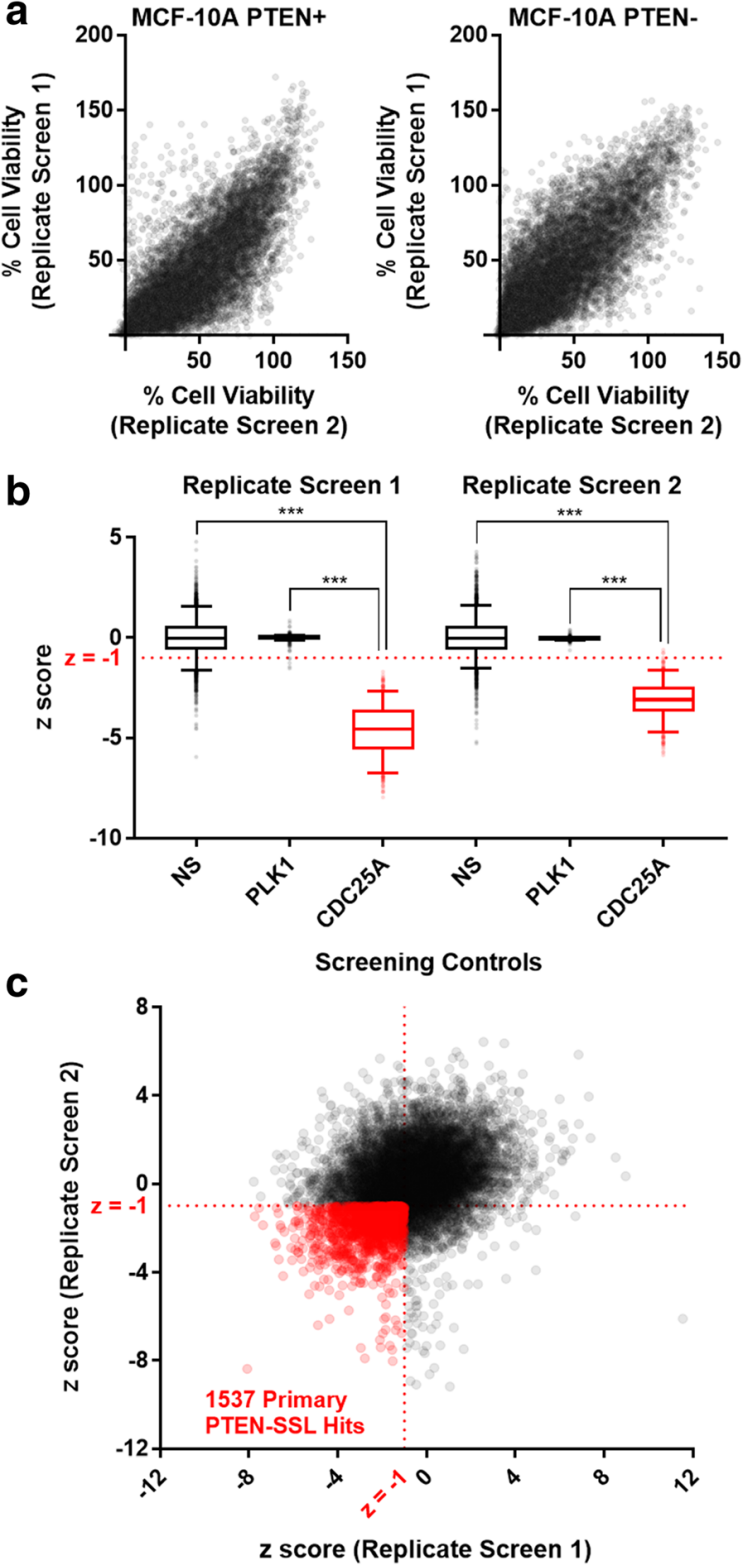
Identification of phosphatase and tensin homolog-synthetic sick/lethal (PTEN-SSL) genes in the MCF-10A cell line. **a** Cell line viability responses to ~ 21,000 short interfering RNA (siRNA) pools in two replicate screens. Each dot represents siRNA pool. **b** The *z* scores for three types of screening control: non-silencing (NS), essential gene (*PLK1*), and PTEN-SSL gene (*CDC25A*). Whiskers indicate 95th and 5th percentiles; ***p < 0.0001 (Wilcoxon rank-sum test). Each dot represents one control siRNA pool. **c** siRNA *z* scores from two replicate screens. Each dot represents one siRNA pool

In the primary screen, a genome-scale siRNA library targeting ~ 21,000 genes was assayed in the MCF-10A PTEN+ and PTEN- cells. We expressed the difference in cell viability between PTEN- and PTEN+ for each siRNA pool as a *z* score based on the distribution of differences in cell viability in the non-targeting (that is, negative) controls (see “Methods”). The *z* scores <0 indicate selective reduction of cell viability or proliferation in the PTEN- cells compared to PTEN+ cells and constitute evidence of PTEN-SSL activity. We carried out two independent PTEN-SSL screens and found screening results to be generally reproducible between replicate screens for both cell lines (Spearman’s rho 0.8, *p* < 0.0001) (Fig. 2a). Importantly, the positive PTEN-SSL control, CDC25A, had mean *z* scores < − 3 in both replicate screens (Fig. 2b). Therefore, we took a *z* score < − 1 in both screens as a non-stringent criterion for identifying candidate PTEN-SSL genes (Fig. 2c). A total of 1537 genes were scored as primary PTEN-SSL hits using this criterion and were prioritized for validation based on the *z* scores (Additional file 1: Table S4).

We used a non-stringent *z* score cutoff for primary hit selection to capture “true positive” PTEN-SSL genes, which would otherwise be excluded by a more stringent cutoff. This is because PTEN synthetic sick genes, by definition, will result in subtle but meaningful reductions in cell viability when silenced by RNAi reagents. However, since off-target gene silencing is well-known to be a considerable source of error in RNAi screens, we expected the 1537 primary hits to include a large number of “false positives” attributable to off-target siRNA effects. Furthermore, because most synthetic sickness and synthetic lethal relationships exist only in certain genetic backgrounds or under specific cellular conditions [36], another subset of primary hits would be PTEN-SSL only in the MCF-10A genetic background and would not be reproduced in other cell lines. We thus followed the primary siRNA screen with a secondary screen designed to filter out the majority of false positive hits. The secondary screen differed from the primary screen in two key ways: (1) the secondary screen was performed using orthogonal RNAi technology, because siRNA off-target effects are unlikely to be reproduced by multiple shRNAs targeting the same gene and (2) the secondary screen was carried out in multiple cell lines to identify genes with possible PTEN-SSL activity in a large proportion of cell lines.

### Secondary shRNA screen in panel of cell lines

**Fig. 3:**
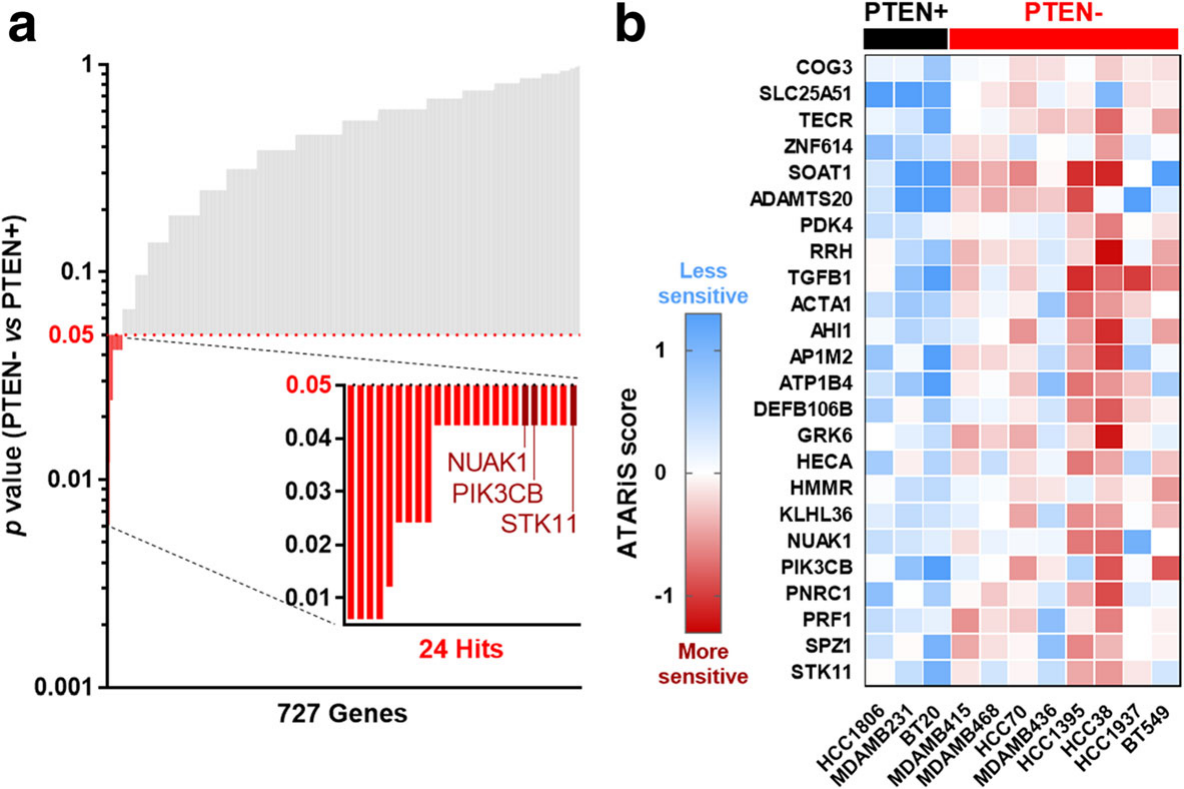
Identification of broad-spectrum phosphatase and tensin homolog-synthetic sick/lethal (PTEN-SSL) genes. **a** The *p* values from the one-sided Wilcoxon rank-sum test for the hypothesis that PTEN- cell lines are more sensitive (lower ATARiS scores) to knockdown of each gene compared to PTEN+ cell lines. Each bar represents one of 727 genes in the secondary screen with computed ATARiS gene solutions. Possible broad-spectrum PTEN-SSL genes were selected using *p* < 0.05 (not corrected for multiple testing) and are shown as red bars. **b** Heat map visualization of the sensitivity (ATARiS scores) of individual cell lines in response to on-target mRNA knockdown of 24 possible broad-spectrum PTEN-SSL genes selected in the secondary screen (red region in **a**)

For the secondary screen, we carried out a pooled lentiviral shRNA dropout viability screen in eight PTEN- and three PTEN+ cell lines (step 2, Fig. 1). All cell lines were screened with a library of 6500 shRNAs targeting ~ 1200 genes, of which ~ 1000 were hits from our primary screen, 9 were putative PTEN-SSL genes reported in other studies but which did not score as hits in the primary screen, and the remaining genes were non-PTEN-SSL controls. Each gene was represented by a pool of 5 to 10 shRNAs. We measured the sensitivity of each cell line to shRNA perturbation by tracking shRNA dropout over four to six population doublings (depending on the doubling time of each cell line). We used the ATARiS algorithm [29] to calculate a sensitivity score for each cell line to each shRNA pool. ATARiS scores were obtained for 727 genes that were deemed to have consistent shRNA effects across the cell lines (ATARiS scores were not obtained for the remainder of the genes, indicating no evidence of PTEN-SSL activity). We then used the one-sided Wilcoxon rank-sum test to identify genes that had lower sensitivity scores in the eight PTEN- cell lines than in the three PTEN+ cell lines (Fig. 3a). A *p* value <0.05 (uncorrected for multiple testing) in the Wilcoxon rank-sum test was used as a non-stringent hit-selection criterion and we identified 24 secondary hits showing evidence of broad-spectrum PTEN-SSL activity across cell lines (Fig. 3b, Additional file 1: Table S9).

As in the primary screen, we used a non-stringent hit criterion with the aim of identifying a reasonably small set of possible broad-spectrum PTEN-SSL genes that would contain a subset of true PTEN-SSL genes. These few true broad-spectrum PTEN-SSL genes would have to be confirmed by subsequent experiments and analyses. Non-stringent statistical criteria have been similarly used for hit selection in synthetic lethal screens in part because of the relatively low validation rate of such screens [37–39]. We used a *p* value-based criterion for hit selection, but, as is typical for high throughput screens, our intent was not to control the family-wise error rate or even to attain a low false discovery rate. Instead, we accepted a very high false discovery rate as a necessary condition for capturing a small number of true discoveries to be identified by subsequent experiments and analyses. We note that this criterion identified phosphatidylinositol-4,5-bisphosphate 3-kinase catalytic subunit beta *(PIK3CB*), a known PTEN-SSL gene [40], as one of the 24 possible broad-spectrum PTEN-SSL hits from the secondary screen. This showed that the chosen criterion was able to identify a true PTEN-SSL gene. Importantly, *PIK3CB* would not have scored as a hit if a more stringent statistical cutoff taking into account multiple testing were used. Thus, other novel, true positive PTEN-SSL genes could potentially be missed if a stringent criterion had been used at this step. As presented next, we subsequently assessed reproducibility of the 24 hits in independent screening datasets and followed this by drug sensitivity experiments using small molecule inhibitors of gene function and by analyses of mutual exclusivity of mutations in tumors.

### PTEN-SSL genes validated in independent RNAi screening datasets

**Fig. 4:**
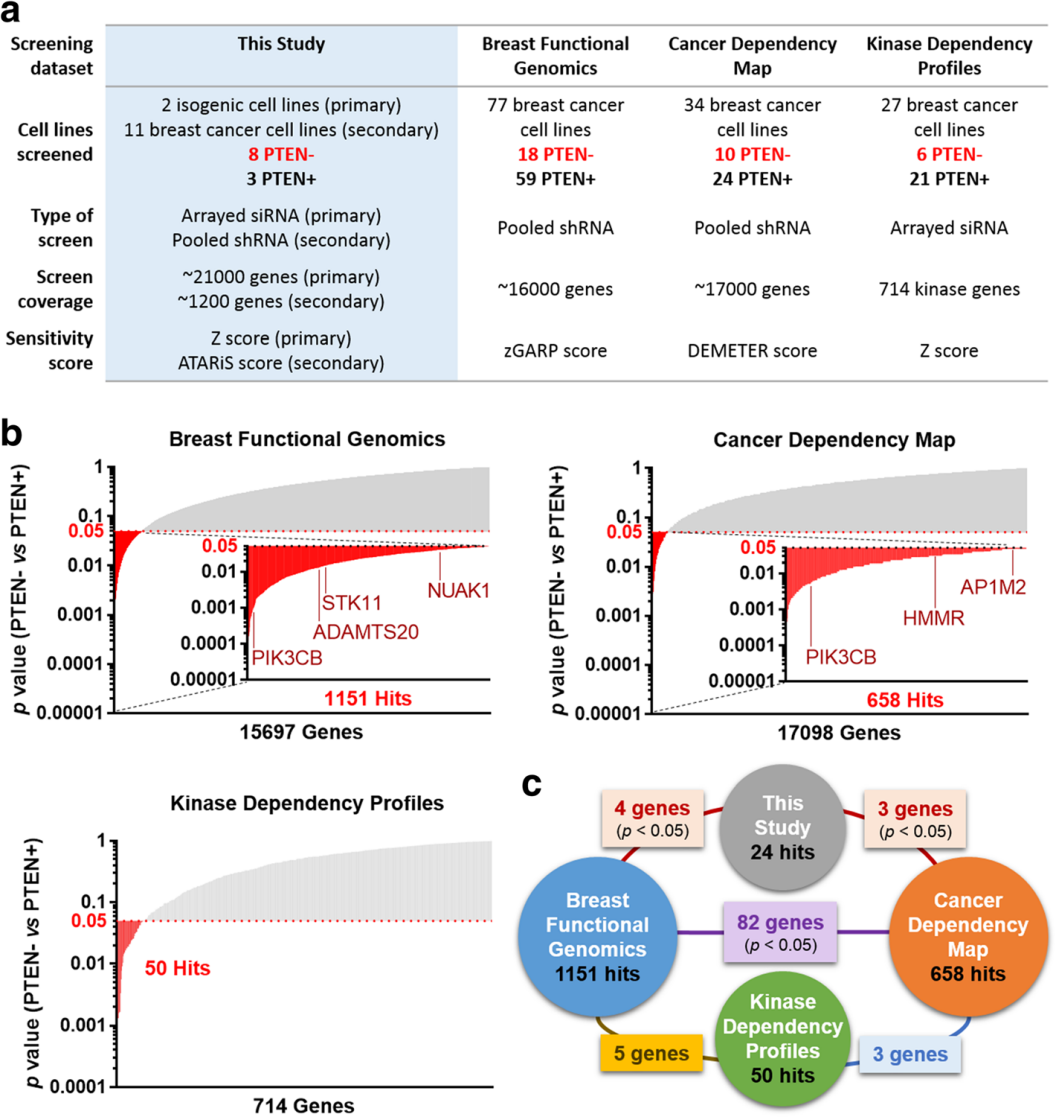
Identification of reproducible broad-spectrum phosphatase and tensin homolog-synthetic sick/lethal (PTEN-SSL) genes. **a** Cmparison of the secondary short hairpin RNA (shRNA) screen described in this study and three independent RNA interference (RNAi) screening datasets selected for reanalysis: Breast Functional Genomics [17], Cancer Dependency Map [19], and Kinase Dependency Profiles [18]. **b** The *p* values from the one-sided Wilcoxon rank sum test for whether PTEN- cell lines were more sensitive (lower ATARiS scores) to knockdown of each gene compared to PTEN+ cell lines. Each bar represents one of 727 genes in the secondary screen that had ATARiS gene solutions. Possible broad-spectrum PTEN-SSL hits were selected using the same criterion (*p* < 0.05, uncorrected for multiple testing) for each screen and are shown as red bars. *PIK3CB*, *NUAK1*, *STK11*, *ADAMTS20*, *AP1M2*, and *HMMR* were found to be hits in our reanalysis of ≥ 1 of the previous screens. **c** Number of overlaps in possible broad-spectrum PTEN-SSL genes that were identified by this study and in reanalysis of three previous screens; *p* values based on one-sided hypergeometric distribution

To date, several high-throughput RNAi screens have been carried out to systematically identify genetic dependencies in large collections of cancer cell lines [17–19, 41–45]. We assessed the reproducibility and generality of our PTEN-SSL hits in the data from three of these previous studies, each of which included > 20 breast cancer cell lines (step 3, Figs. 1 and 4a). The studies we reanalyzed were (1) the Breast Functional Genomics study, comprising genome-scale shRNA screens of 77 breast cancer cell lines [17, 43]; (2) the Cancer Dependency Map study, comprising genome-scale shRNA screens of 501 cancer cell lines, including 34 from breast cancer [19, 42, 44]; and (3) the Kinase Dependency Profiles study, comprising kinome-scale siRNA screens of 117 cancer cell lines, including 27 from breast cancer [18, 41].

We reanalyzed the results of each screen to identify broad-spectrum PTEN-SSL genes by extracting gene-level sensitivity scores reported for each dataset (Fig. 4a). Although each screen was performed using different scoring approaches (zGARP, DEMETER, and *z* scores), increasingly negative scores indicate greater inhibition of cell growth in all cases. We determined the PTEN status of 75 breast cancer cell lines based on previously published reverse-phase protein array data [17], and confirmed that these data correlated strongly with western blot quantification in a subset of 20 cell lines (Spearman’s rho = 0.86, *p* < 0.0001; / Additional file 2: Figure S1). For each gene in each dataset, we selected genes with PTEN-SSL activity using the same method as in our secondary screen, that is by performing the one sided Wilcoxon rank-sum test to identify genes that had lower sensitivity scores in PTEN- cell lines than in PTEN+ cell lines. As in the secondary screen, we used *p* < 0.05 (without correcting for multiple testing) as a non-stringent criterion for selecting hits. This identified 1151, 658, and 50 possible broad-spectrum PTEN-SSL genes from the three studies, respectively (Fig. 4b).

Although no single gene scored as a PTEN-SSL hit across all screens, *PIK3CB* was reproducibly identified as a hit in our screens and in two out of the three reanalyzed screens. In addition, another five genes were identified in reanalysis of one or more of the previous screening studies (Fig. 4b). These genes are (ADAM metallopeptidase with thrombospondin type 1 motif 20 *(ADAMTS20*), (adaptor related protein complex 1 *mu* 2 subunit *(AP1M2*), hyaluronan mediated motility receptor *(HMMR*), NUAK family kinase 1 (*NUAK1*), and serine/threonine kinase 11 (*STK11*). These overlaps in possible broad-spectrum PTEN-SSL genes were statistically significant (*p* < 0.05 as determined using the hypergeometric distribution) (Fig. 4c). The identification of PIK3CB is again an indication that there may be other true positive PTEN-SSL genes among these six genes. We consider these genes candidates for further validation of PTEN-SSL activity in experimental studies and clinical datasets.

### NUAK1 inhibition is PTEN-SSL in breast cancer cell lines

**Fig. 5:**
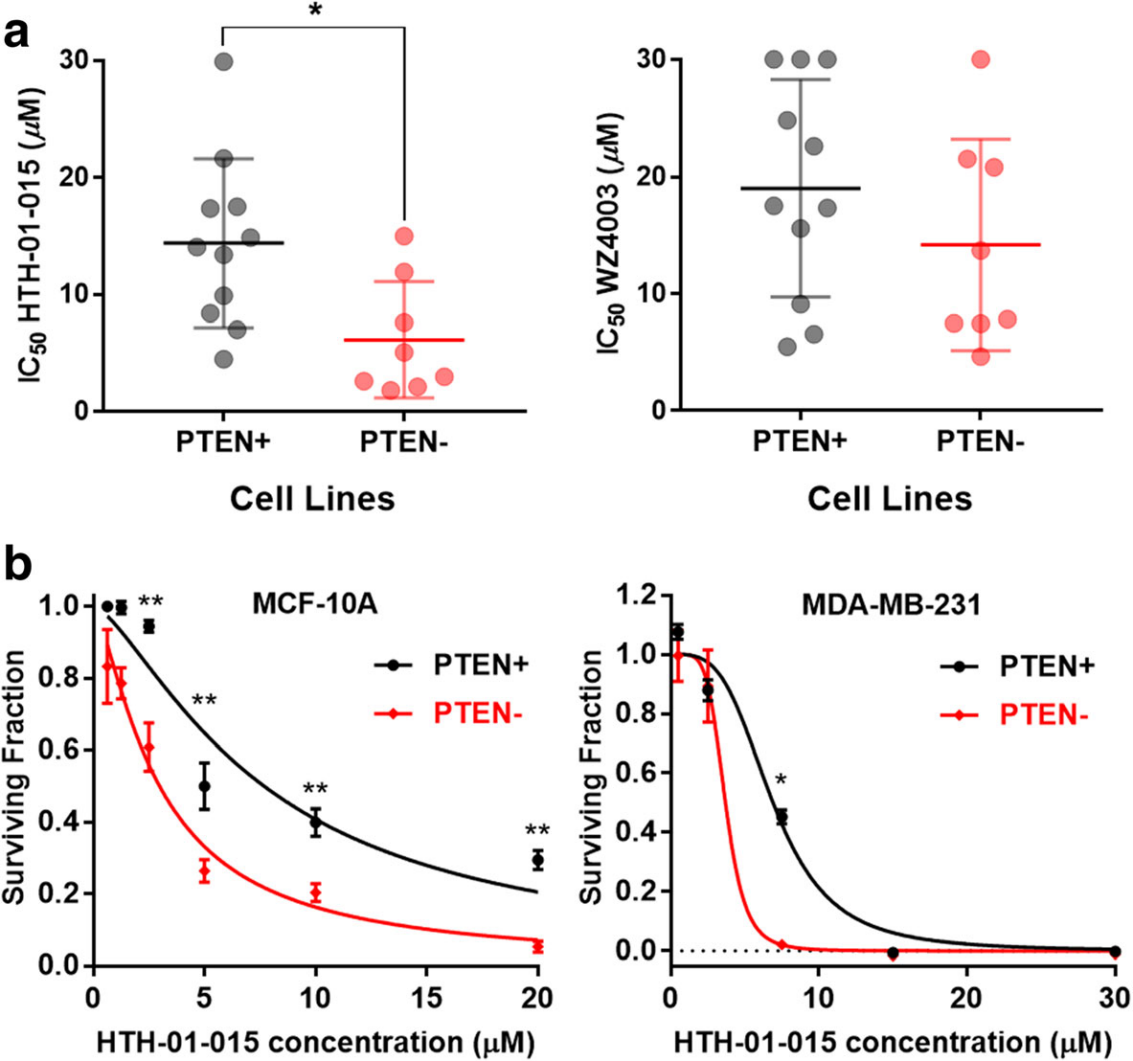
NUAK family kinase 1 (NUAK1) inhibition is phosphatase and tensin homolog-synthetic sick/lethal (PTEN-SSL) in breast cancer cell lines. **a** Sensitivity of 11 PTEN+ and 8 PTEN- breast cancer cell lines to HTH-01-015 and WZ4003, small molecule inhibitors of NUAK1 kinase; *p < 0.05, two-sided Wilcoxon rank-sum test. **b** HTH-01-015 dose-response curves in isogenic PTEN+ and PTEN- models derived from MCF-10A (left) and MDA-MB-231 (right) cell lines;**p < 0.01, *p < 0.05, two-sided *t* test on four biological replicates. IC_50_, concentration required to reduce cell viability by 50%

Two of the broad-spectrum PTEN-SSL genes identified in our screen and shown to be reproducible in reanalysis of data from other studies, *NUAK1*, also known as AMPK-related protein kinase 5 (*ARK5*), and *STK11*, also known as liver kinase B1 (*LKB1*), are functionally related, suggesting that their PTEN-SSL activity may stem from a common mechanism. NUAK1 can be inhibited by commercially available small molecules, and we assessed if two of them, HTH-01-015 and WZ4003 [46], could reduce cell viability selectively in PTEN- cells (step 4, Fig. 1). We compared the sensitivity of PTEN+ and PTEN- cell lines to inhibition by these compounds in 19 breast cancer cell lines (Fig. 5a, Additional file 1: Table S1). PTEN- cell lines were indeed more sensitive to HTH-01-015 treatment than PTEN+ cell lines, with significantly lower IC_50_ values (*p* < 0.05, two-sided Wilcoxon rank-sum test). By contrast, the PTEN- cell lines were not significantly more sensitive to WZ4003. This difference may be due to differing specificity of the two inhibitors: WZ4003 inhibits both NUAK1 and NUAK2 while the activity of HTH-01-015 is more specific to NUAK1 [46].

As a second test of whether PTEN expression determines sensitivity to NUAK1 inhibition by HTH-01-015, we assessed its effects in the MCF-10A and MDA-MB-231 PTEN+/PTEN- isogenic cell lines. In both cases, the PTEN- cell lines were more sensitive to HTH-01-015 than their PTEN+ counterparts (Fig. 5b). Therefore, we concluded that HTH-01-015 selectively targeted PTEN- cell lines and inhibition of NUAK1 has broad PTEN-SSL activity.

### *PTEN* and *STK11* mutations are mutually exclusive in tumors

**Fig. 6:**
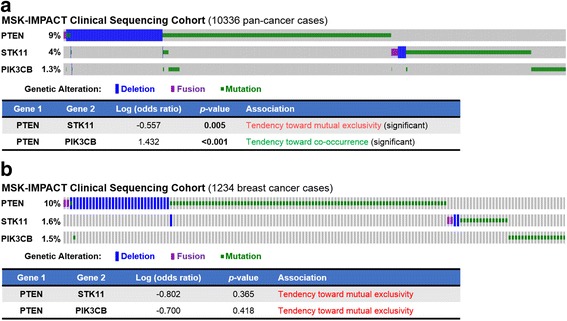
Phosphatase and tensin homolog (PTEN) and serine/threonine kinase 11 (STK11) mutations are mutually exclusive in tumors. OncoPrints show deep (homozygous) deletions, fusions, small insertions and deletions, and non-silent single-base-substitution mutations detected by MSK-IMPACT in all patients studied (**a**) and in the subset of patients with breast cancer within the cohort (**b**). Mutual exclusivity of mutations was determined using odds ratios and the Fisher exact test. Only tumors with mutations are shown

Genes that are SSL with each other would have mutually exclusive loss-of-function mutations in tumors [47]. We therefore used cBioPortal [34, 35] to assess mutual exclusivity between *PTEN* and each of the six broad-spectrum PTEN-SSL genes (*NUAK1*, *STK11*, *PIK3CB*, *HMMR*, *AP1M2*, and *ADAMTS20*). We first examined mutation data in the TCGA breast cancer cohort [32]. None of the associations between mutations in *PTEN* and candidate PTEN-SSL genes was statistically significant (Additional file 3: Figure S2), possibly because of the small sample size. We then examined mutation data in the pan-cancer MSK-IMPACT study, which included > 10,000 patients [33]. Only two of our broad-spectrum PTEN-SSL genes (*STK11* and *PIK3CB*) were assayed in this targeted study. We detected significant *PTEN* SSL effects for *STK11* as indicated by mutual exclusivity of *PTEN* and *STK11* (Fig. 6a). However, *PTEN* and *PIK3CB* mutations tended to co-occur. The analogous analysis of the breast cancer subset of the MSK-IMPACT study found that mutations in both *STK11* and *PIK3CB* tended to be mutually exclusive with PTEN mutations, but was not statistically significant (Fig. 6b). Thus, there is evidence of PTEN-SSL activity of *STK11* in a large pan-cancer patient cohort, and breast cancers within the cohort showed a similar pattern consistent with *PTEN*-*STK11* SSL interaction.

## Discussion

PTEN-SSL genes can be detected through loss-of-function RNAi screens that compare PTEN+ and PTEN- cell lines and several potential PTEN-SSL genes have been reported from such screens [41, 48, 49]. Nevertheless, the lack of overlap in results from these screens is obvious. This is not unique to the search for PTEN-SSL genes, as results from RAS-SSL screens have been similarly divergent [50]. The challenges to reproducibly identifying broad-spectrum SSL vulnerabilities through RNAi screens are well-documented and stem from the genetic complexity of tumor-derived cell lines and from the technical limitations of RNAi screening technologies [36, 50, 51].

We therefore used a screening strategy that started with a comprehensive primary screen in isogenic cell lines and followed up with a hits-focused secondary screen in a cell line panel. We detected 1537 possible PTEN-SSL genes through the primary siRNA screen of isogenic MCF-10A cell lines. In the follow up secondary shRNA screen, 24 genes showed evidence of broad-spectrum PTEN-SSL activity across a panel of 11 genetically diverse breast cancer cell lines. The high PTEN-SSL hit attrition rate was likely driven by genetic variability across the cell lines; in many cases the PTEN-SSL activity was restricted to only one or a small number of cell lines.

We then reanalyzed data from three published RNAi screens carried out in multiple breast cancer cell lines. Through this reanalysis, we identified six genes with reproducible and broad-spectrum PTEN-SSL activities. Among these, *PIK3CB* has previously been reported to be PTEN-SSL while the other five are novel PTEN-SSL genes [40]. *ADAMTS20* encodes a secreted metalloproteinase with increased protein expression in invasive breast carcinoma [52]. *HMMR*, a breast cancer susceptibility gene in *BRCA1* mutation carriers [53, 54], encodes a non-integral hyaluronan receptor that promotes breast cancer migration and invasion in concert with the integral hyaluronan receptor CD44 [55, 56]. *AP1M2* encodes the beta subunit of clathrin-associated adaptor protein complex 1 (AP-1B), which regulates epithelial cell proliferation through its role in protein sorting [57]. Finally, *NUAK1* and *STK11* encode protein kinases, which are functionally related in the AMP-activated protein kinase (AMPK) pathway. STK11 (also known as LKB1) is a well-characterized tumor suppressor that phosphorylates and activates NUAK1 and 12 other members of the AMPK family [58]. We further validated the PTEN-SSL activities of the AMPK pathway members *STK11* and *NUAK1*. We confirmed by small-molecule inhibition by HTH-01-015 that NUAK1 constitutes a novel PTEN-SSL vulnerability across a broad-spectrum of breast cancer cell lines. We also showed that, consistent with PTEN-SSL activity, *PTEN* and *STK11* had mutually exclusive patterns of mutations in the MSK-IMPACT pan-cancer study. This study focused on the mutual exclusivity of somatic loss-of-function mutations. However, the functions of candidate PTEN-SSL genes could also potentially be dysregulated in cancer through epigenetic, transcriptional, translational, or post-translational mechanisms. Thus, mutual exclusivity analyses could be performed on such mechanisms if the relevant data became available.

PTEN, STK11, and NUAK1 have interconnected functions in regulating cell cycle progression and DNA damage repair. Inactivation of either STK11 or PTEN individually accelerates progression through the G1/S checkpoint [59, 60]. Conversely, reconstitution of STK11 or PTEN expression in cells with *STK11* or *PTEN* loss, respectively, induces G1/S arrest [61–63]. Interestingly, NUAK1 functions at the intersection of cell cycle control by STK11 and PTEN. NUAK1 is directly activated by STK11 and indirectly inactivated by PTEN through the AKT pathway [64–66]. NUAK1 activity can either promote cell proliferation or induce cell cycle arrest, depending on whether it is activated by AKT or STK11. In the context of PTEN-deficiency, AKT phosphorylation of NUAK1 at Ser-600 promotes proliferation and invasion in cancer cell lines [64, 67, 68]. Conversely, NUAK1 promotes G1/S arrest through p53 regulation when it is phosphorylated at Thr-211 by STK11 in cells with functional PTEN [58]. NUAK1 overexpression also causes aneuploidy in normal human fibroblast cells, leading to senescence or cell death [69]. In addition to their roles in cell cycle regulation, PTEN, STK11, and NUAK1 are important for DNA repair. All three genes are required for the repair of ultraviolet B-induced DNA damage [70, 71]. In addition, STK11-deficient cells accumulate DNA damage [72], and *PTEN*-deleted cells have defective DNA double-strand break repair mechanisms [73].

**Fig. 7:**
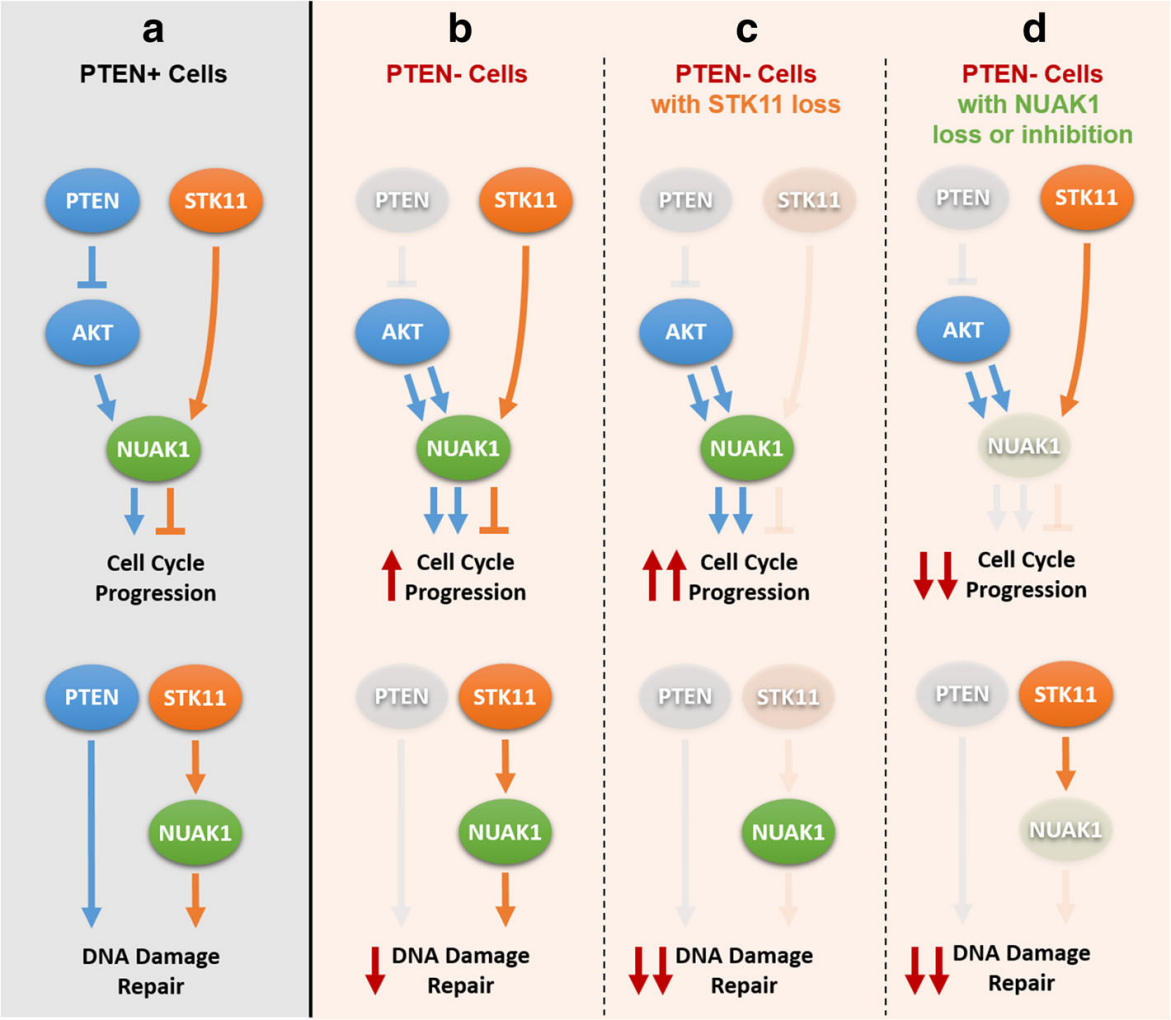
Proposed mechanisms of phosphatase and tensin homolog-serine/threonine kinase 11 (PTEN-STK11) and PTEN-NUAK family kinase 1 (NUAK1) synthetic sickness/lethality. Shown are hypothesized effects of STK11 and NUAK1 loss of function on cell cycle progression (upper panels) and DNA damage repair (lower panels) in PTEN- cells. **a** Roles of selected genes in PTEN+ cells for comparison. **b** In PTEN- cells, AKT signaling is not inhibited by PTEN, leading to increased activation of NUAK1 (double blue arrows), and increased cell-cycle progression (double blue arrows and upward-pointing red arrow in top panel). The PTEN contribution to DNA damage repair is lost (faded gray oval and arrow in bottom panel). **c** In PTEN- cells without STK11, cell cycle progression is even more accelerated, due to loss of STK11-NUAK1 inhibition (faint orange activation and inhibition arcs in top panel). DNA damage repair is further suppressed relative to PTEN- cells with STK11 activity because of lack of activation of NUAK1 by STK11 (faint orange arrows in bottom panel). **d** In PTEN cells without NUAK1, cell cycle progression is arrested (top panel). DNA damage repair is suppressed relative to PTEN- cells with NUAK1 due to loss of NUAK activity (bottom panel)

Therefore, we hypothesize that STK11 and NUAK1 are selectively essential for cell cycle progression and DNA damage repair in PTEN-deficient cancers (Fig. 7). PTEN-deficient cancer cells have increased cell proliferation through accelerated cell cycle progression and also have impaired DNA damage repair. Loss of STK11 function in these cells would reduce its phosphorylation of NUAK1 at Thr-211 and thus deactivate the p53-regulated brake on cell cycle progression (Fig. 7c, top panel, faint orange activation and inhibition arcs compared to Fig. 7b). However, Ser-600 phosphorylation by the PTEN/AKT pathway would continue to promote cell cycle progression through STK11-independent mechanisms (blue arrows in Fig. 7c, top panel). Thus, the net effect of STK11 loss in these cells would be further acceleration of cell cycle. At the same time, STK11 loss would exacerbate the DNA damage repair defect (Fig. 7c, bottom panel). We hypothesize that this combination of effects (accelerated cell cycle progression and increased DNA damage) would lead to death or senescence.

The hypothesized mechanism for PTEN-NUAK1 synthetic sickness/lethality is more direct. Since NUAK1 activation by AKT is involved in driving cell cycle progression caused by loss of PTEN, loss of NUAK1 function would directly reduce cell cycle progression. Consistent with this hypothesis, HTH-01-015 inhibition of NUAK1 in a PTEN-deficient osteosarcoma cell line has been shown to block the G1/S transition [74]. In addition, as with STK11, loss of NUAK1 would further impair DNA damage repair, thus causing further loss of cell viability. In contrast, PTEN-expressing cells would not depend on NUAK1 for cell cycle progression and would retain STK11- and NUAK1-independent DNA damage repair mechanisms. In the future, these hypothesized mechanisms for PTEN-NUAK1 and PTEN-STK11 SSL could be tested by long-term loss-of-function and gain-of-function studies in isogenic PTEN+/− cell lines and in genetically diverse breast cancer cell lines. These would include knocking out *NUAK1* and *STK11* individually in both PTEN+ and PTEN- cell lines using technologies such as CRISPR-Cas9. This would facilitate the study, in detail, of how these PTEN-SSL genes interact with PTEN in regulating cell cycle progression and DNA damage repair. Further chemical screens could also be carried out to identify drugs that would synergize with HTH-01-015 to selectively kill PTEN-deficient cancer cells.

## Conclusions

An RNAi-based functional genomics strategy followed by validation through reanalysis of independent RNAi screens identified six genes with PTEN-SSL activity in a wide range of cell lines: *PIK3CB*, *ADAMTS20*, *AP1M2*, *HMMR*, *STK11*, and *NUAK1.* Two of these, *STK11* and *NUAK1*, function in the AMPK pathway, and we hypothesize that their SSL relationship with *PTEN* stems partly from shared elements of this pathway. In addition, inhibition of NUAK1 with the small-molecule HTH-01-015 was PTEN-SSL in a wide range of cell lines, confirming the broad-spectrum PTEN-SSL activity of *NUAK1*. Our findings support the introduction of therapies targeting NUAK1, including inhibitors such as HTH-01-015, for treatment of PTEN-deficient breast cancers.

## Additional files

Additional file 1: **Table S1.** Growth media for breast cancer cell lines used in study. **Table S2.** PTEN protein abundance in cell lines quantified by western blot. **Table S3.** Classification of breast cancer cell lines as PTEN+ or PTEN- based on PTEN protein abundance. **Table S4.** Primary screen results and hits. **Table S5.** Details of shRNA library used in secondary screen. **Table S6.** shRNA barcode counts from secondary screen in 11 cell lines. **Table S7.** Fold-change in shRNA barcode count for 11 cell lines. **Table S8.** shRNAs used in ATARiS gene solutions, with ATARiS consistency scores per shRNA. **Table S9.** ATARiS gene level scores for 11 cell lines. **Table S10.** Secondary screen results and hits. **Table S11.** Screen results (zGARP scores) from Breast Functional Genomics Dataset. **Table S12.** Screen results (DEMETER scores) from Cancer Dependency Map Dataset. **Table S13.** Screen results (*z* scores) from Kinase Dependency Profiles Dataset. (XLSX 22688 kb)

Additional file 2: **Figure S1.** PTEN protein abundance of breast cancer cell lines. (A) Western blots showing PTEN and actin (loading control) abundance in 19 breast cancer cell lines. (B) Scatter plot of RPPA-measured PTEN abundance reported by Marcotte *et al.* [17] *versus* PTEN abundance that we quantified through densitometric analysis of western blot bands in (A). Cell lines were categorized as PTEN-expressing (in black) or PTEN-deficient (in red) based on PTEN protein abundance. (PNG 201 kb)

Additional file 3: **Figure S2.** Mutual exclusivity analysis in TCGA breast invasive carcinoma cohort. OncoPrints showing deep (homozygous) deletions, fusions, small insertions and deletions, and non-silent single-base-substitution mutations detected by TCGA. Mutual exclusivity of mutations was determined using odds ratios and the Fisher exact test. Only tumors with mutations are shown. (PNG 125 kb)

## Abbreviations

ADAMTS20: ADAM metallopeptidase with thrombospondin type 1 motif 20
AMPK: AMP-activated protein kinase
AP1M2: Adaptor-related protein complex 1 mu 2 subunit
ARK5: AMPK-related protein 5
DMEM: Dulbecco’s modified Eagle’s medium
DMSO: Dimethyl sulfoxide
FACS: Fluorescence-activated cell sorting
HEPES: 2-(4-(2-Hydroxyethyl)piperazin-1-yl)ethanesulfonic acid
HMMR: Hyaluronan-mediated motility receptor
LKB1: Liver kinase b1
MOI: Multiplicity of infection
MTS: 3-(4,5-Dimethylthiazol-2-yl)-5-(3-carboxymethoxyphenyl)-2-(4-sulfophenyl)-2H-tetrazolium
NUAK1: NUAK family kinase 1
PI3K: Phosphatidylinositol 3-kinase (PI3K)
PIK3CB: Phosphatidylinositol-4,5-bisphosphate 3-kinase catalytic subunit beta
PIP_3_: Phosphatidylinositol (3,4,5)-trisphosphate
PTEN: Phosphatase and tensin homolog
RFP: Red fluorescence protein
RNAi: RNA interference
RPMI: Roswell Park Memorial Institute medium
RPPA: Reverse-phase protein array
SDS: Sodium dodecyl sulfate
SDS-PAGE: Sodium dodecyl sulfate polyacrylamide gel electrophoresis
shRNA: Short hairpin RNA
siRNA: Short interfering RNA
SSL: Synthetic sick/lethal
STK11: Serine/threonine kinase 11
TGCA: The Cancer Genome Atlas
