# Supplementary figures and images for: Functional genomics identifies specific vulnerabilities in PTEN-deficient breast cancer
Source: Breast Cancer Res. 2018 Mar 22;20:22. doi: 10.1186/s13058-018-0949-3 (PMC5863852; doi:10.1186/s13058-018-0949-3)

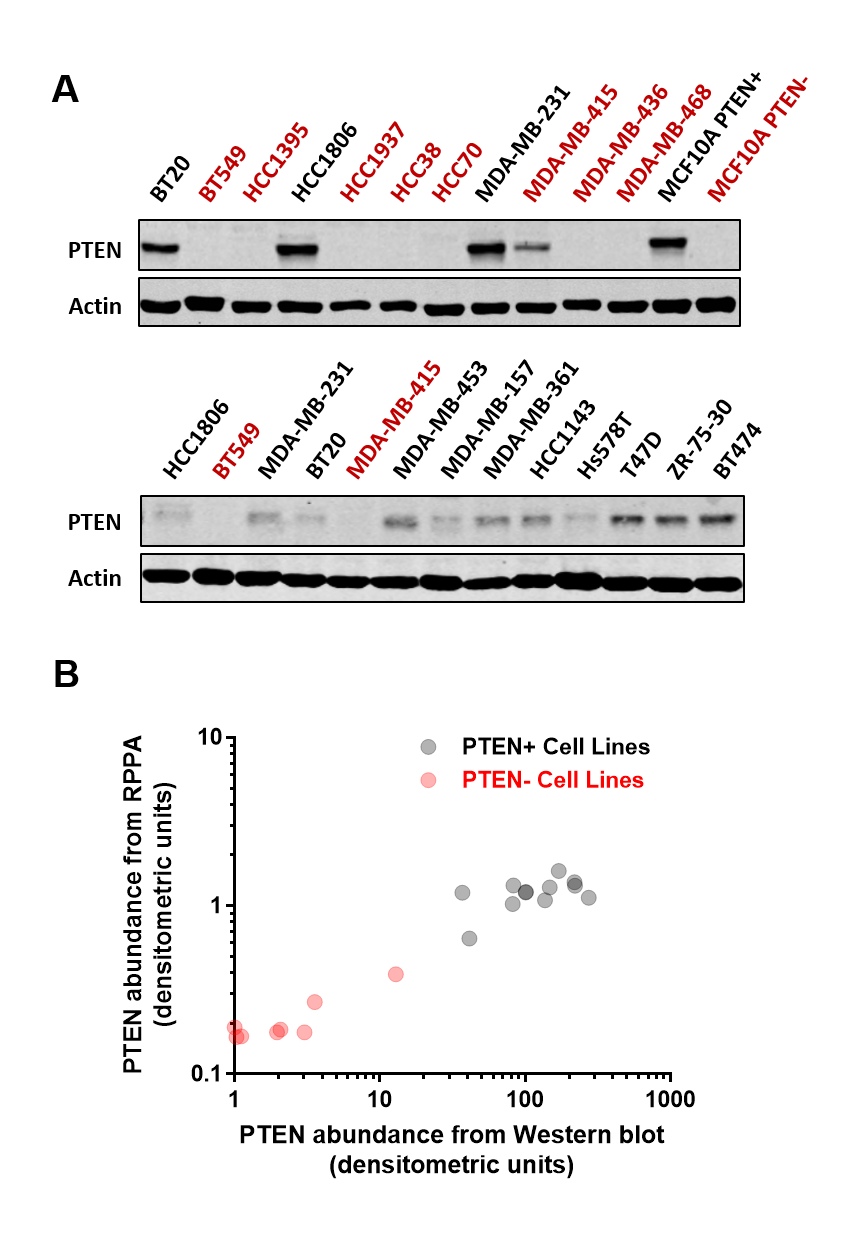

Supplement: Supplementary file 2 — Figure S1. PTEN protein abundance of breast cancer cell lines. (A) Western blots showing PTEN and actin (loading control) abundance in 19 breast cancer cell lines. (B) Scatter plot of RPPA-measured PTEN abundance reported by Marcotte et al. [17] versus PTEN abundance that we quantified through densitometric analysis of western blot bands in (A). Cell lines were categorized as PTEN-expressing (in black) or PTEN-deficient (in red) based on PTEN protein abundance. (PNG 201 kb) [file 13058_2018_949_MOESM2_ESM.png]

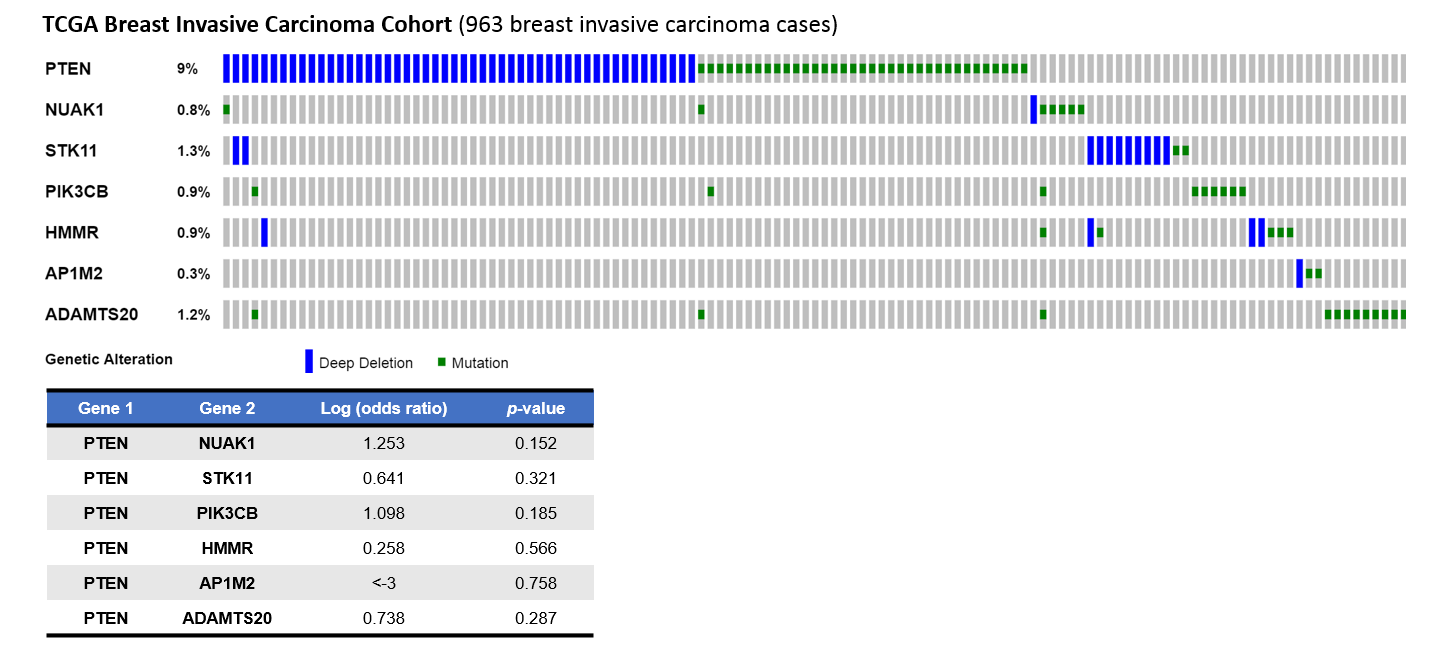

Supplement: Supplementary file 3 — Figure S2. Mutual exclusivity analysis in TCGA breast invasive carcinoma cohort. OncoPrints showing deep (homozygous) deletions, fusions, small insertions and deletions, and non-silent single-base-substitution mutations detected by TCGA. Mutual exclusivity of mutations was determined using odds ratios and the Fisher exact test. Only tumors with mutations are shown. (PNG 125 kb) [file 13058_2018_949_MOESM3_ESM.png]
